# Fixed versus flexible antagonist protocol in women with predicted high ovarian response except PCOS: a randomized controlled trial

**DOI:** 10.1186/s12884-021-03833-2

**Published:** 2021-05-02

**Authors:** Xiu Luo, Li Pei, Fujie Li, Chunli Li, Guoning Huang, Hong Ye

**Affiliations:** 1 Chongqing Key Laboratory of Human embryo Engineering, Chongqing, China; 2 Chongqing Clinical Research Center for Reproductive Medicine, Chongqing, China; 3 Reproductive and Genetic Institute, Chongqing Health Center for Women and Children, Chongqing, China

**Keywords:** Gonadotropin-releasing hormone antagonists, In vitro fertilization, Fixed protocol, Flexible protocol, Number of oocytes retrieved

## Abstract

**Background:**

No previous study directly compares the fixed day-5 initiation versus the flexible initiation of GnRH antagonist administration in IVF/ICSI for those patients who are predicted as high ovarian responders without PCOS. To evaluate whether the number of oocytes retrieved is different by using the two GnRH antagonist protocols in Chinese women with predicted high ovarian response except PCOS.

**Methods:**

A randomized controlled trial of 201 infertile women with predicted high ovarian response except PCOS undergoing in vitro fertilization. Ovary stimulation was performed using recombinant FSH and GnRH antagonists. GnRH antagonist ganirelix (0.25 mg/d) was started either on day 5 of stimulation (fixed group) or when LH was > 10 IU/L, and/or a follicle with mean diameter > 12 mm was present, and/or serum E_2_ was > 600 pg/ml. Patient monitoring was initiated on day 3 of stimulation in flexible group.

**Result(s):**

No significant difference was observed between the fixed and flexible groups regarding the number of oocytes retrieved (16.72 ± 7.25 vs. 17.47 ± 5.88, *P* = 0.421), the Gonadotropin treatment duration (9.53 ± 1.07 vs. 9.67 ± 1.03, *P* = 0.346) and total Gonadotropin dose (1427.75 ± 210.6 vs. 1455.94 ± 243.44, *P* = 0.381). GnRH antagonist treatment duration in fixed protocol was statistically longer than the flexible protocol (6.57 ± 1.17 vs 6.04 ± 1.03, *P* = 0.001). There was no premature LH surge in either protocol.

**Conclusion(s):**

Fixed GnRH antagonist administration on day 5 of stimulation appear to achieve a comparable oocyte retrieved compared with flexible antagonist administration.

**Trial registration:**

NCT02635607 posted on December 16, 2015 in clinicaltrials.gov.

## Background

Gonadotrophin-releasing hormone (GnRH) antagonists have bene widely used for prevention of premature LH surges during controlled ovarian stimulation (COS) before IVF-ET. Recently two meta-analyses have indicated that GnRH antagonist protocol has a similar live-birth rate and significantly improves treatment safety as compared with long GnRH agonist protocols especially for patients with high OHSS risk [1, 2]. Currently, there are two GnRH antagonist protocols (fixed protocol and flexible protocol) with different timing of antagonist initiation in clinical application. It’s evident that the fixed protocol is patient-friendly with less visits and reducing the number of hormone assessment and ultrasound monitoring, and to a certain extent, the flexible protocol may have advantages on decreasing the medicine dose and treatment duration for patients [3].

As to the effectiveness of protocol, in a meta-analysis of four randomized controlled trials (RCTs), the fixed and flexible GnRH antagonist protocols have been found comparable in terms of the number of oocytes retrieved and clinical pregnancy rates, mainly for ovulate women with normal ovarian reserve [3–7]. For patients with high ovarian response, only one RCT including 100 infertile women with polycystic ovarian syndrome (PCOS) showed that the number of oocytes retrieved and good quality embryos in the flexible protocol were more than those in the fixed protocol with similar antagonist dose and less rFSH dose [8].

PCOS is generally regarded as the specific type of infertility patients with high OHSS risk and it has several different phenotypes. Due to various sensitivity of small antral follicles to exogenous FSH, flexible initiation of GnRH antagonist may be more beneficial for women with PCOS. However, no previous study directly compares the two protocols for those patients who are predicted as high ovarian responders without PCOS. Our aim was to assess the effectiveness and efficiency of the fixed versus flexible GnRH antagonist protocol in IVF/ICSI for this group patients.

## Methods

### Patient population

A non-blind randomized controlled trial conducted at the Genetic and Reproductive Institution of Chongqing, China, from January 2016 to July 2017. The study was approved by our Institutional Review Board and registered on the Clinical Trial web site (ClinicalTrials.gov identifier: NCT #02635607).

Inclusion criteria were women aged less than 35 years old, body mass index between 18 and 25 kg/m^2^, a normal menstrual cycle with a range of 21–35 days and at least one of condition was met; 1), the number of oocytes retrieved in previous cycle was more than 15; 2), AMH ≥ 3.52 ng/ml; 3), Antral Follicle Count≥16 [9]. Exclusion criteria were polycystic ovarian syndrome (Rotterdam criteria), a history of low response to FSH treatment, a history of ovariectomy, more than two previous IVF/ICSI, uterine abnormalities which included submucous fibroids, intramural fibroids larger than 3 cm in diameter, uterine malformation, intrauterine adhesions with or without history of previous surgery, more than three previous abortion, and other endocrine disorders.

Two hundred four infertile women were enrolled into the study only once after the Informed consent form was signed. The recruited women were allocated randomly into two groups when Gonadotropin was started on menstrual cycle days 3. Randomization was performed using sealed opaque envelopes prepared by a third party.

### Ovarian stimulation and ART procedures

On the day 3 of menstruation cycle, participants received a fixed dose of 150 IU of recombinant (r) FSH (Follitropin beta, Puregon, MSD, America) for 4 days and individually adjusted thereafter. In group A (the fixed regimen), women received daily 0.25 mg GnRH antagonist (Orgalutran, MSD, America) from simulation day 5 to the day of HCG administration. Women in Group B (the flexible regimen) received daily 0.25 mg GnRH antagonist (Orgalutran, MSD, America) on the day that the diameter of dominant follicle reached 12 mm or estradiol levels>600 pg/ml or LH levels>10 IU/L to the day of HCG administration [10].

When at least three follicles were measured≥17 mm in diameter, patients received their last GnRH antagonist injection in the morning and final follicular maturation was induced the same evening by 250μg rhCG (Ovidrel, Serono, Germany). If there were more than 19 follicles which were ≥ 11 mm in diameter on the day of HCG administration, final follicular maturation was induced the same evening by 0.2 mg GnRH agonist (Diphereline, Ipsen / Decapeptyl, Ferring, Germany). Oocyte retrieval took place 36-38 h after trigger by transvaginal ultrasound-guided double lumen needle aspiration. ICSI would be performed only in cases with severe male factor or previous fertilization failure. Embryo quality was evaluated for all available embryos on day 3 of culture by the experienced embryologist. Embryos graded as grade 1 (6–10 cells, no fragmentation and equal blastomere size) or grade 2 (allowing up to 20% fragmentation) were qualified as good quality embryos. All embryo transfer performed after 72 h after oocyte retrieval by ultrasound guidance. One or two Day3 good quality embryos transferred, and other remaining embryos vitrified for frozen-thawed embryo transfer cycle.

All embryo cryopreservation performed with either circumstance as follow: (i) existed OHSS or high risk evaluated by investigator, (ii) serum progesterone>1.5 ng/ml, (iii) hydrohystera, (iv) agonist trigger. At the following spontaneous menstrual cycle, one or two frozen embryos were thawed every time and transferred 3 days after ovulation until all embryos were transferred. An artificial cycle was used for endometrial preparation in the next menstrual cycle. Estradiol valerate (Progynova, Delpharm Lille, France) at a dose of 4 to 8 mg per day was begun on day 2 or day 3 of the menstrual cycle. When the endometrial thickness reached at least 7 mm, vaginal progesterone gel at a dose of 90 mg per day (Crinone 8% gel, Serono, Germany) was added. Up to 2 day 3 frozen embryos were thawed and transferred 3 days after the start of progesterone.

All patients received luteal support with 90 mg/day progesterone administered intravaginally (Crinone 8% gel, Serono, Germany) starting at the day of oocyte retrieval and continued for at least 12 weeks. Pregnancy test performed for at least 14 days onwards after embryo transfer.

### Hormonal assessments and ultrasound monitoring

Hormonal assessment and ultrasound monitoring were performed on the menstrual cycle day 3(both groups), stimulation day 4 and then daily up to initiation of the antagonist (flexible), initiation day of the antagonist (both groups), the day after antagonist starting (both groups) and the day of hCG trigger (both groups) and additional monitoring decided by investigator as ovarian stimulation response. All blood samples were drawn in the morning before antagonist injection. Serum LH, FSH, hCG, E_2_, and progesterone were assessed by local laboratory at the site. Transvaginal ultrasound performed by skilled ultrasonic technician to measure and count visible follicles by the hospital’s standard procedures for the confirmation of final oocyte maturation triggered as soon as three follicles measuring≥17 mm had been reached. The total numbers of follicles ≥11 mm needed to be visibly counted.

### Outcome measures

The primary endpoint was to assess a difference in the total number of retrieved oocytes between the two groups. The secondary endpoints were the duration and total dose of rFSH and GnRH antagonist, the occurrence of premature LH surges (serum LH>10 IU/L and progesterone>1 ng/ml) and severe OHSS (per World Health Organization criteria, 1973), implantation rate, clinical pregnancy rate, ongoing pregnancy rate and cumulative live birth rate (CLBR). Biochemical pregnancy was defined by serum β-hCG positive 14 days after embryo transfer. Clinical pregnancy was diagnosed by ultrasound detection of gestational sac 2 weeks after positive hCG test. Ongoing pregnancy was defined as a pregnancy with cardiac activity proceeding beyond 12 weeks of gestation. Live birth was defined as delivery of at least one living child at 28 weeks gestation or later with heartbeat and breath. All follow-up periods were 3 years. The CLBRs were calculated by including the first live birth generated during the complete first IVF cycle as the numerator and all women allocated to treatment as the denominator. The estimates of the CLBR assumed that women who did not return for treatment would not have a live birth.

### Statistical analysis

#### Sample size calculation

A sample size of 200 (1:1 allocation) achieved 80% power to detect non-inferiority of the Day-5 fixed-dose regimen as compared with the flexible protocol by a margin at − 3 oocytes retrieved (3 oocytes fewer than the controlled group), using a one-sided, two-sample t-test with Mann-Whitney test adjustment at the significance level at 0.025. The true difference between the means was assumed to be 0.0 and the standard deviation (SD) of both intervention arms to be 6.8. The pre-mature discontinuation rate was set at approximately 15% for this study.

#### Statistical methods

For the primary endpoint, mean and SD on the number of oocytes were presented. The between-group difference and corresponding 95% confidence interval (CI) (Day-5 fixed protocol – flexible protocol) were calculated by using a two-sample t-test under the assumption that the sample data were normally distributed. For the secondary endpoints, the number and percentage of the event were calculated and displayed on categorical variables. Clinical and ongoing pregnancy rates were separately calculated and presented. Between-group comparisons were made by Chi-square test and the corresponding 95%CI will be presented by using Miettinen-Nurminen method if the number of the observed events was at least 4. Mean and SD were summarized for continuous variables in terms of secondary outcome measures. A treatment difference between study groups was made by using two-sample t-test or nonparametric test whenever appropriate.

## Results

**Fig. 1:**
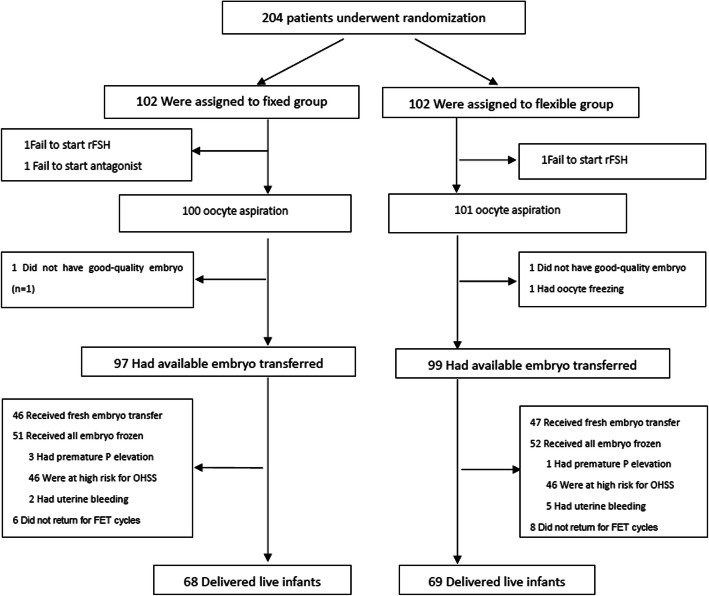
Flow chart on subject disposition

A total of 204 patients participated in the study and were randomized to each two treatment groups. Three patients were discontinued prior to oocyte aspiration due to personal reasons. One hundred patients in fixed protocol group and 101 patients in the flexible protocol group were adhere to the ovarian stimulation protocol and complete the oocyte aspiration. Forty-six in fixed group and 47 in flexible group received fresh embryo transfer (Fig. 1). Finally, 91 patients both in fixed group and flexible group completed all embryo transfer.

The baseline population characteristics of two groups are summarized in Table 1. There were no significant differences between two groups in terms of age, body mass index (BMI), duration of infertility, and type and cause of infertility as well as ultrasonic scanning findings and hormone profiles (*p* > 0.05).

**Table 1:** Baseline characteristics of population

	Fixed group (***n*** = 100)	Flexible group (***n*** = 101)	***p***
**Mean age (year)**	28.9 ± 2.9	28.7 ± 3.3	0.601
**Duration of infertility (year)**	4.8 ± 2.7	4.7 ± 4.2	0.826
**Body mass index (kg/m^2)**	21.1 ± 1.7	21.3 ± 1.9	0.316
**Type of infertility**			
Secondary (%)	57 (57.0)	48 (47.5)	0.205
Primary (%)	43 (43.0)	53 (52.5)	
**Cause of infertility (%)**			
Tubal factor	66 (66.0)	70 (69.3)	0.653
Ovulation dysfunction	1 (1.0)	0	
Endometriosis	0	6 (5.9)	
Male factor	10 (10.0)	11 (10.9)	
Unexplained	11 (11.0)	3 (3.0)	
Multi-factor	12 (12.0)	11 (10.9)	
**AFC**	10.3 ± 2.8	9.9 ± 2.7	0.260
**AMH (ng/ml)**	6.4 ± 2.6	5.8 ± 2.1	0.066
**Baseline sex hormone**			
FSH (IU/L)	5.1 ± 1.1	4.9 ± 1.3	0.144
LH (IU/L)	3.5 ± 1.3	3.3 ± 1.2	0.266
E2 (pg/ml)	29.5 ± 10.8	27.5 ± 9.8	0.189
P (ng/ml)	0.4 ± 0.1	0.3 ± 0.1	0.600

Results of the endpoint analyses are presented in Table 2. The mean (SD) number of oocytes retrieved in the fixed group was 16.72(7.25) which was similar with the mean of 17.47(5.88) in the flexible group. The treatment difference was − 0.75 (95% CI − 2.58 to 1.09; *P* = 0.421). No significant differences were observed between the two groups on the dose of rFSH and duration of stimulation. No premature LH surges were occurred. Treatment duration of GnRH antagonist in fixed protocol group was significantly longer than in flexible group (6.57 ± 1.17 vs. 6.04 ± 1.03, *P* = 0.001).

**Table 2:** Outcomes of ovarian stimulation and embryo culture

	Fixed group (***n*** = 100)	Flexible group (***n*** = 101)	***P***
Duration of rFSH (days)	9.5 ± 1.1	9.7 ± 1.0	0.346
Total amount of rFSH (IU)	1427.8 ± 210.6	1455.9 ± 243.4	0.381
Duration of GnRH antagonist (days)	6.6 ± 1.2	6.0 ± 1.0	0.001
Premature LH rise (LH > 10 IU/L)	1	2	
On Antagonist start day			
E2 (pg/ml)	629.1 ± 294.0	787.7 ± 259.5	<.0001
LH (IU/L)	2.4 ± 2.8	2.7 ± 2.0	0.461
Leading follicle≥12 mm (%)	22 (22.0)	48 (48.5)	<.0001
On hCG trigger day			
E2 (pg/ml)	3373.6 ± 1324.4	3741.0 ± 1099.4	0.034
P (ng/ml)	0.9 ± 0.4	1.0 ± 0.4	0.580
No. of oocytes retrieved	16.7 ± 7.3	17.5 ± 5.9	0.421
No. of MII oocytes	14.9 ± 6.7	15.3 ± 5.5	0.577
No. of good-quality embryos	5.5 ± 3.4	5.7 ± 3.4	0.674

Table 3 shows that implantation rate, clinical pregnancy rate, ongoing pregnancy rate per fresh embryo transfer and cumulative live birth rate per patients were comparable in two groups. Nine patients in flexible group developed the moderate and severe OHSS and 7 patients were observed in fixed group.

**Table 3:** Clinical outcome of embryo transfer and OHSS

	Fixed group (***n*** = 100)	Flexible group (***n*** = 101)	***P***	Difference*[95% CI]
Fresh embryo transfer				
No. of embryo transferred	2.0 ± 0.1	2.0 ± 0	0.182	
Em thickness on the day of ET	9.8 ± 1.4	10.1 ± 1.3	0.130	
Implantation rate (%)	30/91 (33.0)	30/94 (31.9)	0.879	1.05 [−12.44, 14.55]
Clinical pregnancy rate (%)	24/46 (52.2)	24/47 (51.1)	0.915	1.11 [−19.20, 21.42]
Onging pregnancy rate (%)	20/46 (43.5)	17/47 (36.2)	0.472	7.31 [− 12.54, 27.16]
Cumulative live birth rate per patients (%)	68/100 (68.0)	69/101 (68.3)		
OHSS (%)				
Mild	35 (35.0)	38 (37.6)	0.646	
Moderate	5 (5.0)	4 (4.0)		
Severe	2 (2.0)	5 (5.0)		

Treatment difference = Day-5 fixed protocol – flexible protocol

## Discussion

This was the first randomized control trial to compare the clinical outcome of the fixed GnRH antagonist protocol with the flexible protocol in IVF/ICSI for the patients with predicted high ovary response except PCOS, we found no difference in total number of oocytes retrieved in the fixed protocol compared with the flexible protocol. Except the treatment duration of GnRH antagonist in the flexible protocol group was shorter than that in the fixed protocol group, no significant differences were between the two protocols in term of the treatment duration and total dose of rFSH, premature LH surges, implantation, clinical pregnancy, ongoing pregnancy, and cumulative live birth rate.

Previous published studies focused on ovulatory women arrived at the similar outcomes. The early meta-analysis for the patients with normal ovarian response showed us that the outcomes of oocyte retrieval, pregnancy and LH surge suppression were similar between two protocols, whereas the total treatment dose of Gn and GnRH antagonist was significantly less in the flexible protocol group [3]. The original purpose to explore the flexible addition was to delay the initiation timing of GnRH antagonist to reduce the injection but ask for more times of monitoring [11]. Distinctly, our study for women with predicted high ovarian response except PCOS reached the analogous efficiency results as for normal ovary responders, which may be ascribed to similar follicular development during ovarian stimulation for the two groups of patients.

The only 1 RCT for PCOS women revealed the diverse results that the total number of oocytes retrieval and good-quality embryos in the flexible group were remarkably more than those in the fixed group [8]. As the special type of high ovarian responders, the sensibility of follicles to FSH in PCOS patients usually is considered as lower than normal ovarian responder and other high ovarian responders [12]. But in fact, either slow ovarian response or hyperstimulation would easily occur during ovarian stimulation due to inappropriate ovarian stimulation by exogenous FSH, and the heterogeneity of PCOS patients might enhance the probability of uncertain follicle development, so the flexible protocol seems to be more beneficial for PCOS women in clinical outcome, which is also recommended for PCOS women and poor ovarian responders by the clinical consensus on GnRH antagonist protocol in China [13].

Furthermore, the possible reason why there was no significantly difference in the number of oocyte retrieval between two protocols of our study is that the initiation timing of GnRH antagonist was similar. As we known, the fixed protocol is commenced on Gn stimulation day 5 or 6, regardless of follicular development. However, the flexible protocol is administrated only when adequate follicular development (follicular size 12-14 mm) and/or E_2_ production by the developing follicles may give rise to premature LH surge [14]. Anyway, both of standards are not completely evidence based. Although there was significantly difference in the treatment duration of GnRH antagonist in our trial, the actual initiation timing of GnRH antagonist according to the pre-determined initiation standards after the similar rFSH stimulation in the flexible group was very close to stimulation day 5 in the fixed group, and the duration of GnRH antagonist in both groups appeared to be longer than other published trial.

So far, there is not a unified initiation standard of GnRH antagonist in flexible protocol, which is administered just before the expected LH surge mainly relied on the doctor’s experience. In our trial, despite the initiation timing of GnRH antagonist in the flexible group was slightly later and accordingly there were more follicles with diameter of more than 12 mm and higher serum estradiol level on antagonist initiation day, no premature LH surge and few premature LH rises were observed in two groups. we concerned mostly LH surge would result the failure of oocyte retrieval. Apparently, later initiation of GnRH antagonist in the flexible protocol didn’t cause a bad influence on the clinical outcome [15].

Meanwhile, we should realize that the number of available oocytes in ovarian stimulation mostly depends on ovarian reserve and sufficient ovarian stimulation by exogenous FSH. For the patients, the suitable ovarian stimulation including the dose of FSH starting and dose adjustment obviously is more important during the phase of follicle recruitment. Base on the theory of FSH threshold window, unexpected poor ovarian stimulation might be chiefly attributed to insufficient FSH stimulation and earlier GnRH antagonist administration with sufficient stimulation as well [16]. Then the flexible initiation of GnRH antagonist by ultrasound monitoring and serum hormone test has its superiority to avoid the predicament. Undeniably, the fixed protocol has an advantage over the flexible protocol in the aspect of reducing the treatment burden for both patients and doctors.

Certainly, our study has some limitations. First, there is no generally accepted definition of high ovarian responder, which may cause patients heterogeneity especially when sample size is not big enough. Second, RCTs per se frequently have methodological weaknesses, limiting their usefulness in clinical practice. For instance, fixed FSH starting dose may not be sufficient for all the patients, that may reduce the number of oocytes retrieved and influence the outcomes for specific patients. In addition, cumulative pregnancy rate/live birth rate per patients including the frozen–thawed cycles might be more appropriate as key endpoint, also that should be proven with a large sample size. We should notice that, even if the clinical/ongoing pregnancy rate per fresh embryo transfer was a little numerically higher in the fixed protocol, nearly 50% cycle froze all embryos to cancel the fresh transfer in both groups due to high risk of OHSS.

## Conclusions

In conclusion, both fixed and flexible GnRH antagonist protocols can be used in controlled ovarian stimulation for IVF/ICSI for Chinese women with predicted high ovarian response except PCOS. As the precondition of starting dose of 150 IU rFSH, the Day 5 fixed protocol offers a patients-friendly treatment option with competitive effectiveness and efficiency.

## Data Availability

The datasets generated for this study are available on request to the corresponding author.

## Abbreviations

GnRH: Gonadotrophin-releasing hormone
IVF-ET: In vitro fertilization and embryo transfer
COS: Controlled ovarian stimulation
OHSS: Ovarian hyper-stimulation syndrome
RCT: Randomized controlled trial
PCOS: Polycystic ovarian syndrome
FSH: Follicle-stimulating hormone
rFSH: Recombinant follicle-stimulating hormone
ICSI: Intra-cytoplasmic sperm injection
HCG: Human chorionic gonadotrpin
rhCG: Recombinant human chorionic gonadotrpin
LH: Luteinizing hormone
Gn: Gonadotropin
CLBR: Cumulative live birth rate
BMI: Body mass index
